# Клиническая, гормональная и молекулярно-генетическая характеристики 18 случаев нарушения формирования пола (НФП) 46XY, ассоциированных с вариантами в гене SRD5A2

**DOI:** 10.14341/probl13544

**Published:** 2025-12-02

**Authors:** Н. Ю. Калинченко, Н. А. Макрецкая, А. А. Колодкина, М. А. Карева, А. Н. Тюльпаков

**Affiliations:** Национальный медицинский исследовательский центр эндокринологииРоссия; Endocrinology Research CenterRussian Federation; Медико-генетический научный центр имени академика Н.П. БочковаРоссия; Research Centre for Medical GeneticsRussian Federation

**Keywords:** недостаточность 5α-редуктазы II типа, нарушения формирования пола, SRD5A2, 46, XY, дигидротестостерон, deficiency of 5α-reductase type II, disorders of sex development, SRD5A2, 46, XY, dihydrotestosterone

## Abstract

**АКТУАЛЬНОСТЬ:**

АКТУАЛЬНОСТЬ. В структуре нарушений формирования пола (НФП) при кариотипе 46,XY выделяют группу нозологий, обусловленных нарушением синтеза андрогенов, последним этапом которого является превращение тестостерона в более активный андроген дигидротестостерон, что происходит под влиянием фермента 5α-редуктазы II типа (SRD5A2). Дефицит SRD5A2 является редким заболеванием с аутосомно-рецессивным наследованием.

**ЦЕЛЬ:**

ЦЕЛЬ. Дать клиническую и молекулярно-генетическую характеристику 14 новых случаев с подтвержденным молекулярно-генетическим методом дефицитом SRD5A2, а также 4 случаев НФП 46,XY, где были выявлены моноаллельные изменения в гене SRD5A2.

**МАТЕРИАЛЫ И МЕТОДЫ:**

МАТЕРИАЛЫ И МЕТОДЫ. В исследование было включено 310 пациентов с НФП 46,XY. Молекулярно-генетический анализ проводился методом NGS с использованием таргетной панели для мультиплексной амплификации и последующего секвенирования кодирующих последовательностей следующих генов: AKR1C2, AKR1C4, AMH, AMHR2, AR, ARX, ATRX, CBX2, CYB5A, CYP11A1, CYP17A1, DHCR7, DHH, EMX2, ESR2, FGD1, FGF9, FGFR2, FKBP4, FOXF2, FOXL2, HOXA13, HSD17B3, HSD3B2, ICK, LHCGR, LHX1, LHX9, MAMLD1, MAP3K1, MID1, NR0B1, NR5A1, POR, PTGDS, SOX9, SRD5A2, SRY, STAR, SUPT3H, TSPYL1, WNT4, WT1, ZFPM2.

**РЕЗУЛЬТАТЫ:**

РЕЗУЛЬТАТЫ. При молекулярно-генетическом обследовании в гене SRD5A2 было идентифицировано 16 различных вариантов (2 — в нескольких семьях), 4 из которых ранее описаны не были.

**ЗАКЛЮЧЕНИЕ:**

ЗАКЛЮЧЕНИЕ. Проведенное исследование подчеркивает важное значение молекулярно-генетического анализа в дифференциальной диагностике НФП 46,XY.

## Abstract (en)

**INTRODUCTION:**

INTRODUCTION. Among the disorders of sex development (DSD) with karyotype 46,XY, there is a group of diseases caused by defects of androgen synthesis. The last stage of in the synthesis of androgens is the conversion of testosterone into a more active androgen dihydrotestosterone, which occurs under the influence of the enzyme 5α-reductase type II (SRD5A2). SRD5A2 deficiency is a rare disease with autosomal recessive inheritance.

**AIM:**

AIM. To give a clinical and molecular genetic characterization of 14 new cases with confirmed molecular diagnosis of SRD5A2 deficiency, as well as 4 cases of DSD 46,XY, where monoallelic changes in the SRD5A2 gene were detected.

**MATERIALS AND METHODS:**

MATERIALS AND METHODS. The study included 310 patients with DSD 46,XY. Molecular genetic analysis was performed using the NGS method using a targeted panel for multiplex amplification and subsequent sequencing of the coding regions of the following genes: AKR1C2, AKR1C4, AMH, AMHR2, AR, ARX, ATRX, CBX2, CYB5A, CYP11A1, CYP17A1, DHCR7, DHH, EMX2, ESR2, FGD1, FGF9, FGFR2, FKBP4, FOXF2, FOXL2, HOXA13, HSD17B3, HSD3B2, ICK, LHCGR, LHX1, LHX9, MAMLD1, MAP3K1, MID1, NR0B1, NR5A1, POR, PTGDS, SOX9, SRD5A2, SRY, STAR, SUPT3H, TSPYL1, WNT4, WT1, ZFPM2.

**RESULTS:**

RESULTS. By molecular genetic analysis 16 different variants were identified in the SRD5A2 gene (2 in several families), 4 of which had not been previously described.

**CONCLUSION:**

CONCLUSION. The study highlights the importance of molecular genetic analysis in the differential diagnosis of DSD 46,XY.

## ОБОСНОВАНИЕ

Термин нарушения формирования пола (НФП) объединяет группу врожденных состояний, при которых имеют место несоответствие хромосомного, гонадного или анатомического пола [1]. В классификации НФП, предложенной европейскими экспертами, выделяют категорию «НФП 46,XY вследствие нарушения синтеза андрогенов» [1][2]. В данной группе объединены состояния, возникающие при кариотипе 46,XY на относительно позднем этапе формирования пола (после 8-й недели гестации), когда уже произошла закладка яичек, есть секреция антимюллерова гормона (АМГ) клетками Сертоли, ответственного за регрессию мюллеровых протоков, и происходит процесс формирования внутренних и наружных гениталий под влиянием андрогенов. Особое место среди нарушений синтеза андрогенов занимает недостаточность 5α-редуктазы II типа (SRD5A2) — фермента, катализирующего превращение тестостерона в более активный андроген дигидротестостерон. В отличие от других ферментов, катализирующих этапы биосинтеза тестостерона в классических стероидогенных тканях (гонады, надпочечники), SRD5A2 экспрессируется у плода в ткани генитального бугорка и дериватах вольфовых протоков и является необходимым для вирилизации наружных и внутренних гениталий в процессе внутриутробного развития [3]. Подобно некоторым другим формам НФП 46,XY [4][5] при сохранении яичек у пациентов с дефицитом SRD5A2 отмечается развитие маскулинизации в пубертатном периоде. Данный феномен связывают с сохранной функцией изофермента 5α-редуктазы I типа (SRD5A1), который кодируется другим геном и экспрессируется постнатально, таким образом превращая секретируемый гонадами тестостерон в дигидротестостерон [3]. В случае неустановленного диагноза и первоначального выбора женского пола такая маскулинизация может стать причиной смена пола на мужской, что подчеркивает важность максимально ранней диагностики при данном варианте НФП 46,XY.

Дефицит SRD5A2 — редкое заболевание. Ранее нами было описано 3 первых в России пациента с НФП 46,XY вследствие дефицита SRD5A2 [6]. В настоящей публикации мы представляем клиническую и молекулярно-генетическую характеристику 14 новых случаев с подтвержденным молекулярно-генетическим методом дефицитом SRD5A2, а также 4 случая НФП 46,XY, где были выявлены моноаллельные изменения в гене SRD5A2.

## ЦЕЛЬ ИССЛЕДОВАНИЯ

Дать клиническую, гормональную и молекулярно-генетическую характеристику случаев НФП 46,XY, ассоциированных с вариантами в гене SRD5A2.

## МАТЕРИАЛЫ И МЕТОДЫ

Место проведения. ФГБУ «НМИЦ эндокринологии» Минздрава России.

Время исследования. Январь 2015 — декабрь 2019 гг.

Анализ данных проведен на основании предоставленных выписок для проведения генетического исследования пациентов с НФП 46,XY и у пациентов, проходивших обследование в Институте детской эндокринологии ФГБУ «НМИЦ эндокринологии» Минздрава России за период с 2015 по 2019 гг.

Популяция: пациенты от 0 до 17 лет с НФП 46,XY.

Критерии включения: несоответствие между хромосомным мужским полом 46,XY и строением наружных и/или внутренних половых органов.

Критерии исключения: наличие синдромальной патологии с множественными пороками развития.

Способ формирования выборки из изучаемой популяции: сплошной способ формирования выборки.

## Дизайн исследования

Одноцентровое ретроспективное одновыборочное исследование, включившее 310 пациентов с НФП 46,XY.

Пациентам проводилось комплексное обследование, включающее: оценку строения наружных половых органов по Прадеру, ультразвуковое исследование малого таза, паховых каналов, органов мошонки, исследование гормонального статуса — ЛГ, ФСГ, тестостерон, ДГТ, Э2.

Молекулярно-генетический анализ проводился в лаборатории отделения наследственных эндокринопатий. Геномную ДНК выделяли из лейкоцитов периферический крови стандартным методом (набор Pure Link, Genomic DNA Mini Kit, Life Technologies, США). Методика ДНК-анализа для молекулярно-генетического анализа применялся метод NGS. Использовалась разработанная в отделении наследственных эндокринопатий панель праймеров для мультиплексной ПЦР и секвенирования с применением технологии Ion Ampliseq™ Custom DNA Panel (Life Technologies, США). Панель праймеров «Нарушения формирования пола» охватывает кодирующие области следующих генов: AKR1C2, AKR1C4, AMH, AMHR2, AR, ARX, ATRX, CBX2, CYB5A, CYP11A1, CYP17A1, DHCR7, DHH, EMX2, ESR2, FGD1, FGF9, FGFR2, FKBP4, FOXF2, FOXL2, HOXA13, HSD17B3, HSD3B2, ICK, LHCGR, LHX1, LHX9, MAMLD1, MAP3K1, MID1, NR0B1, NR5A1, POR, PTGDS, SOX9, SRD5A2, SRY, STAR, SUPT3H, TSPYL1, WNT4, WT1, ZFPM2. Подготовка библиотек проводилась в соответствии с рекомендациями производителей. Секвенирование осуществлялось на полупроводниковом секвенаторе PGM (Ion Torrent, Thermo Scientific, США) или Illumina MiSeq (Illumina, США). Биоинформатическая обработка результатов секвенирования проводилась с помощью программных модулей Torrent Suite 4.2.1 (Ion Torrent, Life Technologies, США) или Genome Analysis ToolKit (GATK) ver. 4.1.2.0 (Broad Institute, Cambridge, MA, USA). Для аннотирования вариантов нуклеотидной последовательности использовался пакет программ ANNOVAR ver. 2018Apr16. После анализа полученных данных проводилось подтверждение полученных мутаций на секвенаторе Genetic Analyzer Model 3130 (Life Technologies, США). Оценка влияния выявленных вариантов нуклеотидной последовательности на сплайсинг in silico производилась с помощью веб-ресурса SpliceAI (https://spliceailookup.broadinstitute.org/). Оценка патогенности вариантов нуклеотидной последовательности проводилась согласно международным и российским рекомендациям [7][8]. Нумерация кодирующей последовательности гена дана по референсу NM_000348.3 (http://www.ncbi.nlm.nih.gov/genbank).

## РЕЗУЛЬТАТЫ

В исследование было включено 310 пациентов с НФП 46,XY за период с 2015 по 2019 гг. По результатам проведенных молекулярно-генетических исследований биаллельные нуклеотидные замены в гене SRD5A2 были выявлены в 14 случаях, что составило 4,5% от общего числа пациентов с НФП 46,XY. Моноаллельные нуклеотидные замены в гене SRD5A2 были выявлены в 4 случаях, что составило 1,3% от общего числа пациентов с НФП 46,XY.

## Пациенты с биаллельными вариантами в гене SRD5A2

Из 14 пациентов с биаллельными мутациями в гене SRD5A2 при рождении 10 были зарегистрированы в женском паспортном поле (степень вирилизации по Прадеру 1–2) и 4 — в мужском паспортном поле (степень вирилизации по Прадеру 2–3). Поводом для обследования являлось выявление гонад в паховых областях при женском фенотипе в 8 случаях, двойственное строение наружных половых органов при рождении (у 3) или проявления маскулинизации в пубертатном периоде (у 2). У 5 пациентов первоначально был ошибочно диагностирован синдром резистентности к андрогенам (СРА), и у 1 — ВДКН. Возраст верификации дефицита SRD5A2 варьировал от 4 мес. до 17 лет. 4 из 14 пациентов были сибсами.

Результаты исследования ДГТ были доступны в 6 случаях. У всех 6 пациентов в возрасте от 6 мес. до 14 лет обследование было проведено на фоне пробы с хорионическим гонадотропином человека (ХГЧ), и стимулированные уровни Т, ДГТ и соотношение Т/ДГТ у них варьировали от 7,5 до 12,3 нмоль/л, 1,31 до 1,1 нмоль/л и от 5,7 до 11,3 соответственно.

При молекулярно-генетическом обследовании было идентифицировано 12 различных вариантов в гене SRD5A2, среди которых у 11 пациентов (8 семей) были обнаружены гомозиготные варианты, и у 3 — компаунд-гетерозиготные варианты. 3 из 12 вариантов (p.Leu130Pro, p.Cys222Tyr и c.281+2T>A) ранее описаны не были. Среди 12 вариантов было 3 нонсенс-варианта (p.Gln6*, p.Arg103*, p.Tyr136*), 6 миссенс-вариантов (p.Leu55Pro, p.Leu130Pro, p.Cys222Tyr, p.Arg246Gln, p.Tyr91Asp, p.Tyr235Phe), 2 варианта, влияющих на сплайсинг (c.281+2T>A, c.589G>A), и 1 делеция со сдвигом рамки считывания (p.Leu152Tyrfs*8). В соответствии с критериями патогенности 6 вариантов (p.Gln6*, p.Arg103*, p.Tyr136*, p.Leu152Tyrfs*8, c.281+2T>A, c.589G>A) были классифицированы как патогенные, 5 (p.Leu55Pro, p.Cys222Tyr, p.Arg246Gln, p.Tyr91Asp, p.Tyr235Phe) — как вероятно патогенные, и 1 (p.Leu130Pro) — как вариант неопределенного значения.

2 из 12 вариантов были выявлены минимум в 2 неродственных семьях: p.Leu55Pro (2 пациента, 3 хромосомы) и c.589G>A (3 пациента, 6 хромосом) (табл. 1).

**Table:** Таблица 1. Пациенты с биаллельными вариантами в гене SRD5A2 Ж — женский пол, м — мужской пол, ж/м — смена пола, калькулятор патогенности - http://calc.generesearch.ru/ HGMD_ID — ссылка на вариант в базе данных HGMD https://www.hgmd.cf.ac.uk

	Пациент, пол воспитания	Фенотип	Генотип	Тип мутации	Оценка патогенности	HGMD_ID
1	С.Б. ж	Прадер 1, яички в половых губах	c.[ 589G>A];[ 589G>A]p.[ (Glu197Lys)];[ (Glu197Lys)]	Сплайсинг	P (PM2, PVS1, PP4)	CM1922241 [6][15]
2	Д.М. ж	Прадер 2, яички в паховых каналах	c.[ 16C>T];[ 16C>T]p.[ (Gln6*)];[ (Gln6*)]	Нонсенс	P (PM2, PVS1, PP4)	CM033029 [16]
3	И.Д. ж	Прадер 1, яички в половых губах	c.[ 164T>C];[ 164T>C]p.[ (Leu55Pro)];[ (Leu55Pro)]	Миссенс	LP (PM2, PP3, PM3, PP5, PP4)	CM093695 [17]
4	К.С. ж	Прадер 1, яички в половых губах	c.[ 389T>C];[ 389T>C]p.[ (Leu130Pro)];[ (Leu130Pro)]	Миссенс	VUS (PM2, PP3, PP4)	новый
5	П.А. ж	Прадер 2, яички в паховых каналах	c.[ 164T>C];[ 665G>A]p.[ (Leu55Pro)];[ (Cys222Tyr)]	МиссенсМиссенс	LP (PM2, PP3, PM3, PP5, PP4);LP (PM2, PP3, PM3, PP5, PP4)	CM093695 [17];новый
6	Б.О. ж	Прадер 2, яички в паховых каналах	c.[ 589G>A];[ 589G>A]p.[ (Glu197Lys)];[ (Glu197Lys)]	Сплайсинг	P (PM2, PVS1, PP4)	CM1922241 [6][15]
7	С.Р. м	Нет информации	c.[ 737G>A];[ 737G>A]p.[ (Arg246Gln)];[ (Arg246Gln)]	Миссенс	LP (PM2, PP3, PP1, PM3, PP5, PP4)	CM920644 [14][25]
8	Х.Н. ж	Прадер 2, яички в паховых каналах	c.[ 281+2T>A];[ 281+2T>A]	Сплайсинг	P (PM2, PVS1, PP4)	новый
9	Х. ж	Прадер 2, яички в паховых каналах	c.[ 281+2T>A];[ 281+2T>A]	Сплайсинг	P (PM2, PVS1, PP4)	новый
10	Ц.А. ж/м	Прадер 2, яички в паховых каналах	c.[ 271T>G];[ 453delC]p.[ (Tyr91Asp)];[ (Leu152Tyrfs*8)]	МиссенсСдвиг рамки	LP (PM2, PP3, PM3, PP4);P (PM2, PVS1, PP4)	CM931411 [18];CD136856 [19]
11	Ч.М. м.	Прадер 2, яички в половых губах	c.[ 704A>T];[ 704A>T]p.[ (Tyr235Phe)];[ (Tyr235Phe)]	Миссенс	LP (PM2, PP3, PM3, PP5, PP4)	CM030966 [20]
12	Ч.А. м.	Прадер 2, яички в половых губах	c.[ 704A>T];[ 704A>T]p.[ (Tyr235Phe)];[ (Tyr235Phe)]	Миссенс	LP (PM2, PP3, PM3, PP5, PP4)	CM030966 [20]
13	Ч.Е. ж	Прадер 1, яички в паховых каналах	c.[ 307C>T];[ 408C>A]p.[ (Arg103*)];[ (Tyr136*)]	НонсенсНонсенс	P (PM2, PVS1, PP4);P (PM2, PVS1, PP4)	CM021698 [21];CM196262 [22]
14	Ц.С. ж	Прадер 2, яички в половых губах	c.[ 589G>A];[ 589G>A]p.[ (Glu197Lys)];[ (Glu197Lys)]	Сплайсинг	P (PM2, PVS1, PP4)	CM1922241 [6][15]

## Пациенты с моноаллельными вариантами в гене SRD5A2

Из 4 пациентов с моноаллельными мутациями в гене SRD5A2 при рождении 3 были зарегистрированы в мужском паспортном поле (степень вирилизации по Прадеру — 3–4) и 1 в женском паспортном поле (степень вирилизации по Прадеру — 2). Результаты исследования ДГТ не были доступны ни в одном из случаев.

При молекулярно-генетическом обследовании было идентифицировано 4 различных гетерозиготных варианта в гене SRD5A2, 2 из которых (p.Pro181Leu и p.Y136*) ранее были описаны у пациентов с биаллельными вариантами в гене SRD5A2, а 2 других (c.675G>A p.Gly226= и c.591G>A p.Glu197=) являются новыми. Патогенность последних 2 вариантов пока не ясна. Обе однонуклеотидные замены по предсказанию не приводят к изменению кодонов (синонимичные варианты), однако при анализе in silico могут оказывать эффект на сплайсинг.

## ОБСУЖДЕНИЕ

Хотя в литературе имеются и более ранние описания семейных случаев НФП 46,XY с проявлениями поздней вирилизации [9], впервые доказательства связи синдрома с дефицитом 5α-редуктазы были представлены Walsh с соавт. в 1974 г. [10]. Авторы описали семейный случай НФП 46,XY с женским фенотипом при рождении и вирилизацией в пубертате, где было документировано снижение уровня ДГТ в крови и нарушение перехода Т в ДГТ в фибробластах генитальной кожи in vitro [12]. В том же году Imperato-McGinley с соавт. представили данные обследования 24 пациентов НФП 46,XY из 13 семей из географического изолята в Доминиканской Республике, у которых фенотипически отмечалось близкое к женскому строение наружных половых органов при рождении и выраженная маскулинизация в пубертатном периоде с 100%-ным выбором мужской психосоциальной ориентации [11]. В пользу дефицита 5α-редуктазы свидетельствовали повышение соотношений 3α,5β-этиохоланолона к 3α,5α-андростерону и 3α,5β-этиохоландиола к 3α,5α-андростандиолу в моче, а также уровней указанных метаболитов после введения in vivo меченого тритием [³H] тестостерона [11].

Andersson с соавт. представили доказательства связи заболевания именно с 5α-редуктазой II типа, выявив с использованием Southern блоттинга делецию гена SRD5A2 у пациентов из географического изолята в Папуа — Новой Гвинее, при этом было продемонстрировано, что ген SRD5A1 оставался интактным [12]. Кодируемый геном SRD5A2 одноименный белок содержит 254 аминокислотных остатка и имеет 50%-ную гомологию с 5α-редуктазой I типа (SRD5A1) [12].

Thigpen с соавт. картировали ген на хромосоме 2p23.1 [13]. В другом исследовании та же группа авторов установила молекулярную причину дефицита SRD5A2 в ранее описанном географическом изоляте в Доминиканской Республике, которой оказалась миссенс-мутация в кодоне 246 [14].

В настоящее время у пациентов с данной формой НФП 46,XY описано более 150 патогенных вариантов в гене SRD5A2 (https://www.hgmd.cf.ac.uk). В обследованной нами когорте 11 вариантов (9 — в группе биаллельных мутаций, 2 — моноаллельных) были описаны ранее [14–23]. Большинство вариантов являются редкими и не описаны в популяционных базах данных или представлены там с крайне низкой частотой. Исключением является вариант c.737G>A Arg246Gln, затрагивающий CpG сайт и встречающийся в базе данных gnomAD с частотой 0,0001 (https://gnomad.broadinstitute.org/). Данный вариант был выявлен нами в гомозиготном состоянии у пациента с III ст. вирилизации по Прадеру, который был зарегистрирован при рождении в мужском поле. Похожий фенотип при данной мутации описан и другими авторами [25]. Еще у 2 сибсов с III ст. вирилизации по Прадеру (№11, 12) был выявлен гомозиготный вариант c.704A>T p.Tyr235Phe, для которого в литературе есть описания случаев как с женским, так и мужским выбором пола при рождении [20]. У обследованных нами пациентов с женским фенотипом при рождении определялись как нонсенс-, так и миссенс-варианты, причем в одном случае гомозиготная мутация была классифицирована как VUS. В целом, большинство авторов указывают на отсутствие корреляции генотип-фенотип при дефиците SRD5A2 [26]. Между тем отдельные исследования у пациентов с «легким» фенотипом подтверждают связь клинических проявлений и частично сохранной функции фермента. Так, Nordenskjöld и Ivarsson описали семейный случай гипоспадии с сохранной фертильностью при дефиците SRD5A2, обусловленном компаунд-гетерозиготными мутациями Gly196Ser и His231Arg, для которых была показана остаточная функциональная активность in vitro [27].

Отдельно следует остановиться на варианте c.589G>A, который в настоящем исследовании был выявлен нами в гомозиготном состоянии у 3 пациентов с женским фенотипом при рождении (№1, 6, 14). Примечательно, что все 3 пациента были этнически буряты. Данный вариант был описан при дефиците SRD5A2 у пациента из Китая [15] и представлен в гетерозиготном состоянии с общей частотой 8,0E-6 в базе gnomAD, где он встречается только в южноазиатской и восточноазиатской группах (https://gnomad.broadinstitute.org/). Проведенный нами ранее анализ частоты носительства данного варианта в Республике Бурятия показал, что он составляет 1:51, при этом расчетная частота НФП 46,XY вследствие дефицита SRD5A2 составила 1:20 000 [24]. По предсказанию последовательности транскрипта вариант c.589G>A должен приводить к замене кодона глютаминовой кислоты на лизин в положении 197 (p.Glu197Lys). Между тем нами было показано, что замена c.589G>A влияет на сплайсинг, что было доказано in silico и в эксперименте in vitro. Полученные данные позволили реклассифицировать вариант как влияющий на сплайсинг и обосновать его патогенность (PM2, PVS1, PP4 http://calc.generesearch.ru) [24].

ДНК-анализ является в настоящее время основным методом диагностики дефицита SRD5A2. Ранее нами было показано, что соотношение концентраций Т/ДГТ в крови не является надежным диагностическим критерием [5]. Ряд авторов также указывают на трудности диагностики при использовании данного показателя, особенно у детей раннего возраста [28][29]. В настоящем исследовании измерение соотношения Т/ДГТ было доступно у 6 пациентов, отмечена его значительная вариабельность, что подтверждает нецелесообразность измерения ДГТ при проведении дифференциальной диагностики НФП 46,XY.

Нами отдельно выделена группа из 4 пациентов, у которых были выявлены моноаллельные варианты в гене SRD5A2. Фенотипически у 3 пациентов строение наружных гениталий при рождении было ближе к мужскому (Прадер 3), и у 1 соответствовало женскому. У пациента с женским фенотипом в пубертате отмечались проявления вирилизации, что свидетельствует в пользу дефицита SRD5A2. Выявленные 2 синонимичных варианта (табл. 2, пациенты 2, 3) не встречаются в базе данных gnomAD, и данные анализа in silico (https://spliceailookup.broadinstitute.org/) показывают, что оба варианта могут влиять на сплайсинг. Таким образом, связь НФП 46,XY с дефицитом SRD5A2 в этой группе пока не может считаться доказанной. В перспективе планируются дополнительные исследования (полногеномное секвенирование) для поиска возможного второго варианта, располагающегося в интронах или регуляторной области гена.

**Table:** Таблица 2. Пациенты с моноаллельными вариантами в гене SRD5A2

Пациент	Фенотип	Генотип	Тип мутации	Оценка патогенности	HGMD_ID
1. Б.С. м	Прадер 4, яички в мошонке	c.[ 542C>T];[ ?]p.[ (Pro181Leu)];[ ?]	Миссенс	LP (PM2, PP3, PM3, PP5, PP4)	CM054125 [23]
2. М.М. ж	Прадер 2, двусторонний паховый крипторхизм	c.[ 675G>A];[ ?]p.[ (Gly226=)];[ ?]	?	VUS	новый
3. Г.Г. м	Прадер 4, односторонний паховый крипторхизм	c.[ 591G>A];[ ?]p.[ (Glu197=)];[ ?]	?	VUS	новый
4. З.В. м	Прадер 3, двусторонний паховый крипторхизм	c.[ 408C>A];[ ?]p.[ (Tyr136*)];[ ?]	Нонсенс	P (PM2, PVS1, PP4)	CM196262 [22]

## ЗАКЛЮЧЕНИЕ

Таким образом, дефицит 5α-редуктазы II типа не является частым вариантом НФП 46,XY, как считалось ранее. Однако его своевременная диагностика позволяет выбирать мужской пол воспитания, независимо от степени нарушения строения наружных половых органов при рождении. До настоящего времени отсутствуют надежные гормональные маркеры заболевания и проведение молекулярно-генетической диагностики является единственным методом верификации дефицита SRD5A2. С учетом обнаружения эффекта основателя среди бурятов целесообразно рекомендовать проведение поиска мутации c.589G>A в гене SRD5A2 при рождении ребенка с НФП 46,XY как первую линию диагностики в данной этнической группе.

## ДОПОЛНИТЕЛЬНАЯ ИНФОРМАЦИЯ

Источники финансирования. Публикация настоящей работы поддержана благотворительным фондом филантропии КАФ, программа «Альфа-Эндо».

Конфликт интересов. Авторы декларируют отсутствие явных и потенциальных конфликтов интересов, связанных с содержанием настоящей статьи.

Участие авторов. Все авторы одобрили финальную версию статьи перед публикацией, выразили согласие нести ответственность за все аспекты работы, подразумевающую надлежащее изучение и решение вопросов, связанных с точностью или добросовестностью любой части работы.

Родители пациентов добровольно подписали информированное согласие на публикацию персональной медицинской информации в обезличенной форме в журнале «Проблемы эндокринологии».
